# Congenital Malformations in Singleton Infants Conceived by Assisted Reproductive Technologies and Singleton Infants by Natural Conception in Tehran, Iran

**DOI:** 10.22074/ijfs.2018.5415

**Published:** 2017-10-14

**Authors:** Ramin Mozafari Kermani, Mansoureh Farhangniya, Seyed Abolhassan Shahzadeh Fazeli, Pezhman Bagheri, Mahnaz Ashrafi, Ahmad Vosough Taqi Dizaj

**Affiliations:** 1 Health Metrics Research Center, Iranian Institute for Health Sciences Research, ACECR, Tehran, Iran; 2 Human and Animal Cell Bank, Iranian Biological Resource Center (IBRC), ACECR, Tehran, Iran; 3 Department of Genetics, Reproductive Biomedicine Research Center, Royan Institute for Reproductive Biomedicine, ACECR, Tehran, Iran; 4 Department of Molecular and Cellular Biology, Faculty of Basic Sciences and Advanced Technologies in Biology, University of Science and Culture, Tehran, Iran; 5 Noncommunicable Diseases Research Center, Fasa University of Medical Sciences, Fasa, Iran; 6 Department of Endocrinology and Female Infertility, Reproductive Biomedicine Research Center, Royan Institute for Reproductive Biomedicine, ACECR, Tehran, Iran; 7 Department of Reproductive Imaging, Reproductive Biomedicine Research Center, Royan Institute for Reproductive Biomedicine, ACECR, Tehran, Iran

**Keywords:** Assisted Reproductive Technology, Congenital Malformations, Embryo Transfer, *In Vitro* Fertilization, Sperm Injections, Embryo Transfer

## Abstract

**Background:**

Multiple pregnancies occur more frequently in assisted reproductive technology (ART) compared to normal conception (NC). It is known that the risk of congenital malformations in a multiple pregnancy are higher than single pregnancy. The aim of this study is to compare congenital malformations in singleton infants conceived by ART to singleton infants conceived naturally.

**Materials and Methods:**

In this historical cohort study, we performed a historical cohort study of major congenital malformations (MCM) in 820 singleton births from January 2012 to December 2014. The data for this analysis were derived from Tehran’s ART linked data file. The risk of congenital malformations was compared in 164 ART infants and 656 NC infants. We performed multiple logistic regression analyses for the independent association of ART on each outcome.

**Results:**

We found 40 infants with MCM 29 (4.4%) NC infants and 14 (8.3%) ART infants. In comparison with NC infants, ART infants had a significant 2-fold increased risk of MCM (P=0.046). After adjusting individually for maternal age, infant gender, prior stillbirth, mother’s history of spontaneous abortion, and type of delivery, we did not find any difference in risk. In this study the majority (95.1%) of all infants were normal but 4.9% of infants had at least one MCM. We found a difference in risk of MCMs between *in vitro* fertilization (IVF) and intracytoplasmic sperm injection (ICSI). We excluded the possible role of genotype and other unknown factors in causing more malformations in ART infants.

**Conclusion:**

This study reported a higher risk of MCMs in ART singleton infants than in NC singleton infants. Congenital heart disease, developmental dysplasia of the hip (DDH), and urogenital malformations were the most reported major malformations in singleton ART infants according to organ and system classification.

## Introduction

A highly contested subject exists for assisted reproductive technologies (ART) and congenital malformations in infants (1). The higher risk of congenital malformations in ART infants in comparison with infants from normal conception (NC) is one of the greatest concerns for these children (2). The authors of the present paper have previously assessed 400 ART infants for the incidence of major congenital malformations (MCM) without a control group. We determined a 7% frequency of disorders, which was 2-3% higher than infants in the public population. There was no significant difference between two groups of intracytoplasmic sperm injection (ICSI) and *in vitro* fertilization (IVF) in that study (P=0.08) (3). In our previous study, 309 (43%) infants were from multiple pregnancies. There was a greater risk of congenital malformations in the multiple pregnancies compared to the single pregnancies (4). The incidence of congenital malformations in ART infants compared with NC infants in a number of studies showed a rate of congenital malformations in ART infants 2 times higher than the control group (5,6). There was no difference observed between ICSI and IVF in the incidence of congenital malformations (7,9). 

Others reported no difference in a comparison between ART infants and NC infants (1,5,10,12). Unlike the above studies, some studies were solely carried out to determine the differences in the incidence of congenital malformations in single ART infants compared to single NC infants. This group of studies reported a greater incidence of congenital malformations in ART infants compared to NC infants (5,13,15). The incidence of congenital malformations was equal between ICSI and IVF groups in some studies (3,5,7,9,11,13). Although, in one paper, there were more congenital malformations in the ICSI group (1.58%) compared to the IVF group (1.11%, P=0.052) (12). The contradictory results mentioned in the above articles have led us to study the presence of MCM in single ART infants and compare the results with single NC infants. In addition, we compared the incidence of MCM between ICSI and IVF infants. 

## Materials and Methods

This was a historical cohort study of MCM in 820 births from January 2012 to December 2014. We compared the incidence of MCMs among 168 ART infants (exposed group) to 652 NC infants (non-exposed group). We assessed approximately 4 NC infants for each ART infant. This retrospective record linkage cohort study used the following data set. The ART database (exposed group) was obtained from the Child Health and Development Research Center (CHDRC) which is a subset of the Iranian Academic Center for Education, Culture, and Research (ACECR). All mothers underwent treatment at Royan Institute for Reproductive Biomedicine (RI-RB). The exposed and unexposed infant data were gathered from CHDRC. We have defined MCM according to the International Classification of Disease-11 (ICD-11). 

In Tehran, the CHDRC is the center which issues health certificates for children from birth until 16 years of age. Hence, numerous infants from various districts of Tehran voluntary are referred to this center in order to obtain full visiting rights and are followed for several years. We obtained demographic information and the results from two visits that included infant’s sex, mother’s age, reproductive technology, mothers’ history of stillbirth and abortion, type of delivery, and complete medical records. The inclusion criteria consisted of infants followed by CHDRC after two examinations at the center (during the first 6 months of age and between 6 and 18 months of age); no major genetic disease in the infant’s family history; no exposure to X-ray radiation; no abdominal trauma during pregnancy; resident of Tehran; first born; singleton child; no drug or medicine usage by the mother during pregnancy; and no parental family relationships. 

### Statistical analysis

We used descriptive statistics to determine the prevalence of MCMs in both the singleton ART and NC groups. Multiple logistic regression analyses (backward model) with SPSS-21 software were used to estimate the odds ratio (OR) with 95% confidence interval (CI) to establish a relationship between ART for each outcome. Independent variables consisted of type of delivery, infant’s sex, mother’s age, reproductive technology, prior stillbirth, and history of spontaneous abortion. We entered mothers’ age, mother’s history of stillbirth and abortion, and type of delivery to the model to determine if these were confounding factors. For all of the mentioned outcomes, we performed stratified analyses to control for the bias of confounding effects. In the present study, all MCM both groups were assessed according to ICD-11 criteria (i.e., infants that needed surgery up to the age of 1 year and developed a defect in organ function). The Research Ethics Committee of ACECR and Royan Institute Institutional Review Board approved this study. 

## Results

Of 820 singleton infants identified, we selected 168 ART infants (exposed group) and 652 NC infants (control, non-exposed group) from the CHDR Center during 2012 to 2014. The prevalence rate of MCM in singleton ART and singleton NC groups, a comparison of MCM between the exposed and unexposed singleton infants, and a separate comparison of MCM for IVF and ICSI singleton infants (Table 1). This table shows the variables as maternal age and infant’s sex in ART infants compared with NC infants. There were no statistically significant differences in the rate of malformations for age groups and infant’s sex. In the two groups, NC mothers had an average age of 28 years (28.6 ± 4.4); while for ART mothers it was 31.2 years (31.2 ± 4.8). There were 51% boys and 49% girls in both groups. According to ICD-11, hypospadias, inguinal hernia, severe PDA+VSD, stenosis of the lacrimal duct until age one year, urethral reflux more than grade 3, hydronephrosis, undescended testis (until one year of age), severe amblyopia, club foot, developmental dysplasia of the hip (DDH) that required surgery, and rickets resistant to conventional therapy were considered major malformations. MCM analysed according to specific risk factors in both singleton ART and singleton NC infants (Table 1), in addition to MCM for IVF and ICSI. 

MCM analysed according to specific risk factors in both singleton ART and singleton NC infants (Table 2). We found a nonsignificant, increased risk of MCM (P=0.052, 95% CI: 1.01-3.78). When we entered confounding factors-history of abortion during pregnancy, prior stillbirths, and delivery methods in the both univariate and multivariate models, the effects on significance and risk of MCM was P=0.047 with a 95% CI of 1.01-3.78. We sorted the prevalence of MCM in both groups of infants (Table 3).

**Table 1:** The effects of demographic and other variables on quality of life (QoL) in infertile couples


Variable		NC	ART			Total
			ART	ICSI	IVF	
		n (%)	n (%)	n (%)	n (%)	n (%)

All infants		652 (80)	168 (20)	134 (15.9)	34 (4.1)	820 (100)
Maternal age (Y)						
	<35	531 (81.4)	124 (73.8)	100 (74.6)	24 (70.6)	655 (79.4)
	>35	121 (18.6)	44 (26.2)	34 (25.4)	10 (29.4)	165 (20.6)
Delivery						
	Normal	110 (16.8)	5 (3)	3 (2.2)	2 (5.9)	115 (14)
	Cesarean	542 (83.2)	163 (97)	131 (97.8)	32 (94.1)	705 (86)
Sex						
	Boy	337 (51.7)	81 (48.2)	68 (50.7)	13 (38.3)	418 (51)
	Girl	315 (48.3)	87 (51.8)	66 (49.3)	21 (61.7)	402 (49)
History of abortion						
	No	542 (83.1)	23 (13.7)	12 (9)	11 (32.4)	565 (69)
	>1	110 (16.9)	145 (86.3)	122 (91)	23 (67.6)	255 (31)
History of stillbirth						
	No	644 (98.8)	164 (97.6)	132 (98.5)	32 (94.1)	808 (98.5)
	>1	8 (1.2)	4 (2.4)	2 (1.5)	2 (5.9)	12 (1.5)
Major congenital malformations (MCM)						
	No	623 (95.6)	154 (91.6)	124 (92.5)	30 (88)	777 (94.8)
	Yes	29 (4.4)	14 (8.4)	10 (7.5)	4 (12)	43 (5.2)


ICSI; Intracytoplasmic sperm injection, ART: Asisted reproducive technologies, and IVF: In vitro fertilization.

**Table 2:** Rate of major congenital malformations (MCM) compared in singleton assisted reproductive technology (ART) and singleton normal conception (NC) infants


Variable		MCM		OR (95% CI)(Crude)	P value(Crude)	OR (95% CI)(Adjusted)^*^	P value(Adjusted)
		No	Yes				

Reproductive technology					0.059		0.047
	Normal	623 (95.6%)	29 (4.4%)	Reference		Reference	
	ART	154 (91.7%)	14 (8.3%)	1.89 (0.98-3.66)		1.89 (1.01-3.66)	
Sex					0.87		0.79
	Boy	397 (95%)	21 (5%)	Reference		Reference	
	Girl	380 (94.5%)	22 (5.5%)	1.09 (0.59-2.02)		1.08 (0.58-2.01)	
Maternal age (Years)					0.56		0.73
	<35	622 (95 %)	33 (5%)	Reference		Reference	
	>35	155 (93.9%)	10 (6.1%)	1.22 (0.59-2.52)		1.14 (0.54-2.37)	
History of stillbirth					0.48		0.74
	No	766 (94.8%)	42 (5.2%)	Reference		Reference	
	>1	11 (91.7%)	1 (8.3%)	1.96 (0.21-13.15)		1.41 (0.17-11.38)	
History of abortion					0.129		0.65
	No	540 (95.6%)	25 (4.4%)	Reference		Reference	
	>1	237 (92.9%)	18 (7.1%)	1.64 (0.88-3.06)		1.21 (0.53-2.76)	
Delivery					0.25		0.34
	Normal	107 (97.3%)	3 (2.7%)	Reference		Reference	
	Cesarean	670 (94.4%)	40 (5.6%)	2.13 (0.65-7.01)		1.80 (0.53-6.04)	


*; Adjusted for all variables in Table 1. OR; Odds ratio and CI; Confidence interval.

**Table 3:** Prevalence of major congenital malformations (MCM) in singleton assisted reproductive technology (ART) and singleton normal concep­tion (NC) infants


Reproductive technologies	NC	ART
Disease	n (%)	n (%)

Congenital heart disease (PDA+VSD)	-	3 (1.9)
Developmental dysplasia of the hip (DDH)	-	2 (1.2)
Urethral stenosis	5 (0.8)	2 (1.2)
Hypospadias	6 (0.9)	1 (0.6)
Undescended testis	6 (0.9)	2 (1.2)
Lacrimal duct stenosis	6 (0.9)	1 (0.6)
Fusion labia	2 (0.3)	-
Craniosynostosis	-	2 (1.2)
Hydronephrosis+urethral reflux	-	1 (0.6)
Cleft lip and palate	-	1 (0.6)
Hermaphroditism	2 (0.3)	-
Down syndrome	1 (0.1)	-
Club foot	1 (0.1)	-
Total malformations	29 (4.4)	15 (1.82)
Total infants	652 (79.5)	168 (20.5)


## Discussion

Approximately 20% of pregnant women, were 35 years or older, which was relatively similar in both the ART and NC groups. However, between the two IVF and ICSI groups, there were more mothers aged 35 years or older in the IVF group. In terms of method of delivery, 86% had a cesarean section, which was elevated in the control group. In the present study, there were 14 out of 168 (8.3%) ART infants and 29 out of 652 (4.4%) NC infants with MCM. This implied that the number of congenital disorders in singleton ART infants was two times that of singleton NC infants, however this finding was not statistically significant in univariate analysis (OR=1.95, 95% CI: 1.01-3.78, P=0.052). Analysis of the confounding factors-history of abortion, prior stillbirths, and delivery methods according to univariate and multivariate models showed a significance level of P=0.047 and 95% CI: 1.01-3.78 for the risk of MCM. The above findings supported the results of four review studies and meta-analysis until 2013 with regards to single ART infants. These four papers reported higher numbers of major congenital disorders in single ART infants compared with single NC infants (4, 8, 9, 15).

Studies reported a two-fold greater risk of emergence of congenital disorders in single ART infants compared with single NC infants (5, 6, 14, 15). The above findings indicated that the possibility of major congenital disorders in single ART infants was higher than single NC infants. In contrast, Moses et al. (1), Yan et al. (12), and Bassiouny et al. (10) did not report any significant difference between control and single ART groups. In the present study, 8.9% of all single ART infants had one major congenital disorder. The ratio of ICSI to IVF was twice (8.2 vs. 32.6%) which indicated a significant difference (P=0.047). The majority of studies in this field did not report any significant differences between single ICSI and single IVF in terms of congenital disorders (4, 8, 9). In a study by Yan et al. (12) there were 1.58% congenital disorders in the ICSI group compared to 1.11% in the IVF group, which showed a significant difference (P=0.052, OR=1.42, 95% CI: 0.99-2.03) (12). The history of abortion in both groups of ART mothers (89.6%) was much higher than NC mothers (17.1%). There was no significant difference between the two ART groups in history of abortion (P=0.89). After adjustments for maternal age (ART mothers become pregnant approximately 5 years later than NC mothers) and infant's sex, stillbirth, abortion, and type of delivery, we found no difference in risk (OR=1.95, P=0.047, 95% CI: 1.01-3.78).

In the current study, the most common disorders in single ART infants were congenital cardiac diseases (1.9%), genitourinary system disorders (3.5%), DDH (1.2%), lacrimal duct obstruction (0.6%), craniosynostosis (1.2%), and lip and palate cleft (0.6%). The most prevalent disorders were related to the cardiovascular and urogenital systems. In NC infants, the most common disorders were related to the urogenital system and lacrimal duct obstruction. In our previous study with ART infants, we observed a higher frequency with congenital cardiac diseases, urogenital system disorders, and musculoskeletal disorders (3).

The prevalence of systems and organs involved in congenital disorders varied amongst different studies. Perhaps the reasons for these differences were due to careful and continuous examinations, differences in the numbers of cases, and use of different equipment for examinations and diagnoses (i.e., kidney or brain sonography). Yan et al. (12) reported that the cardiovascular system (0.29%), central nervous system (0.2%), and limb disorders (0.13%) were the most common involved sites, whereas Hansen et al. (5) stated that musculoskeletal disorders (3.8%), urogenital disorders (2.7%), and cardiovascular disorders (1.3%) were the most frequently observed. Midrio et al. (2) reported a high prevalence of anorectal disorders (OR=13.3, 95% CI: 4-39.6) in ART infants.

## Conclusion

Some studies reported a slightly increased risk of MCM in ART infants. We have observed a higher risk of MCM in ART singleton infants compared to NC. In singleton ART infants, congenital heart disease, DDH, and urogenital malformations were the most commonly observed MCMs. More cardiovascular and endocrine malformations were observed in singleton ART infants compared to singleton NC infants. Therefore, we recommend that ART infants should undergo more precise examinations with regards to the above body systems.
